# Endoplasmic reticulum stress-induced cellular dysfunction and cell death in insulin-producing cells results in diabetes-like phenotypes in *Drosophila*

**DOI:** 10.1242/bio.046524

**Published:** 2019-12-20

**Authors:** Hiroka Katsube, Yukiko Hinami, Tatsuki Yamazoe, Yoshihiro H. Inoue

**Affiliations:** Department of Insect Biomedical Research, Research Center for Insect Advanced Studies, Kyoto Institute of Technology, Matsugasaki, Kyoto, Japan, 606-0962

**Keywords:** ER chaperone, UPR, Apoptosis, JNK pathway, *Drosophila*

## Abstract

The destruction of pancreatic β cells leads to reduced insulin secretion and eventually causes diabetes. Various types of cellular stress are thought to be involved in destruction and/or malfunction of these cells. We show that endoplasmic reticulum (ER) stress accumulation in insulin-producing cells (IPCs) generated diabetes-like phenotypes in *Drosophila*. To promote the accumulation of extra ER stress, we induced a dominant-negative form of a *Drosophila* ER chaperone protein (Hsc70-3^DN^) and demonstrate that it causes the unfolded-protein response (UPR) in various tissues. The numbers of IPCs decreased owing to apoptosis induction mediated by caspases. The apoptosis was driven by activation of Dronc, and subsequently by Drice and Dcp-1. Accordingly, the relative mRNA-expression levels of *Drosophila* insulin-like peptides significantly decreased. Consistent with these results, we demonstrate that glucose levels in larval haemolymph were significantly higher than those of controls. Accumulation of ER stress induced by continuous Hsc70-3^DN^ expression in IPCs resulted in the production of undersized flies. Ectopic expression of Hsc70-3^DN^ can induce more efficient ER stress responses and more severe phenotypes. We propose that ER stress is responsible for IPC loss and dysfunction, which results in diabetes-related pathogenesis in this *Drosophila* diabetes model. Moreover, inhibiting apoptosis partially prevents the ER stress-induced diabetes-like phenotypes.

## Abstract (precis)

**Summary:** A new *Drosophila* diabetes model, where ER stress accumulation and apoptosis is induced in IPCs, showed diabetes-like phenotypes including reduced *dilp* mRNA, increased glucose concentration in hemolymph and growth inhibition.

## INTRODUCTION

Diabetes is a group of metabolic diseases wherein patients show hyperglycaemia, which is a condition of elevated blood sugar level. This disease is classified into three principal types: type 1 diabetes (T1D), type 2 diabetes (T2D) and gestational diabetes mellitus (Collares et al., 2013). Among them, T1D is believed to be an autoimmune disease characterized by inflammatory responses, which results in progressive destruction of pancreatic β-cells. This cell damage causes insulin deficiency and deregulation of glucose metabolism. Exogenous insulin therapy is the only treatment for T1D (Okur et al., 2017; Rani and Bhadada, 2017). To develop substantially more effective therapeutic agents, it is quite important to identify factors involved in the pathogenesis and understand the mechanisms underlying the onset of T1D. The onset of diabetes is caused by the destruction or dysfunction of pancreatic β-cells (Keskinen et al., 2002; Ferrannini et al., 2010; Sreenan et al., 1999; Ize-Ludlow et al., 2011). Recently, it has been suggested that autoimmune responses and various types of cellular stresses, especially endoplasmic reticulum (ER) stress, cause β-cell destruction or malfunction (O'Sullivan-Murphy and Urano, 2012; Tersey et al., 2012; Lombardi and Tomer, 2017). Other previous reports showed defects in the expression of some unfolded-protein response (UPR) mediators in pancreatic cells from diabetic patients and in mouse models of the disease. It was also suggested that a functional UPR helps preserve pancreatic cells (Engin et al., 2013). Furthermore, the importance of ER stress-induced apoptosis in the development of diabetes has been suggested (Oyadomari et al., 2002). Although previous studies suggested that a connection exists between the onset of diabetes and ER stress, the mechanism whereby ER stress or its response (UPR) trigger β-cell destruction has not been clarified.

Excessive accumulation of misfolded or unfolded proteins (exceeding the folding capacity of chaperones) causes a stress condition called ER stress and activates the UPR. Three principal branches of the UPR have been identified (Walter and Ron, 2011); each branch is regulated by a different sensor transmembrane protein, namely inositol-requiring enzyme 1 (IRE1), double-stranded RNA-activated protein kinase-like ER kinase (PERK) and activating transcription factor 6 (ATF6). These proteins sense unfolded protein accumulation and activate the transcription of genes encoding ER chaperone proteins, such as GRP78. Cell death is induced in cases of excessive stress accumulation that cannot be managed by the UPR. ER stress-induced apoptosis is associated with many diseases, such as neurodegenerative diseases, atherosclerosis and diabetes (Kaufman, 2002). Tissues in which large amounts of secreted proteins are synthesized (like pancreatic β-cells) are particularly sensitive to ER stress induction (Boot-Handford and Briggs, 2010). Thus, excessive ER stress or attenuation of the UPR in IPCs potentially causes destruction or functional inhibition of the cells, resulting in reduced insulin secretion and eventually causing diabetes. However, it remains uncertain whether ER stress and attenuation of the UPR promotes development of the disease. Extensive studies at the individual level are essential to clarify the causal relationship.

The fruit fly *Drosophila melanogaster* has served as an excellent model for many diseases during the past decade, including metabolic diseases (Allocca et al., 2018; Ugur et al., 2016; Inoue et al., 2018; Yamaguchi, 2018). Insulin, its receptor and the insulin-signaling pathway are highly conserved between mammals and *Drosophila* (Teleman, 2010). In *Drosophila*, eight types of insulin-like peptides (Dilp1–8) have been identified, although no insulin-like growth factors (IGFs) have been found (Brogiolo et al., 2001; Broughton et al., 2005; Garelli et al., 2012; Colombani et al., 2012). Dilp1–7 bind as ligands to a unique insulin receptor, InR, and trigger the well-conserved insulin-signaling pathway. Among them, Dilp2, Dilp3 and Dilp5 are expressed in 14 insulin-producing median neurosecretory cells (m-NSCs) in the brain. Especially, Dilp2 is a principal circulating insulin in flies and is essential for maintaining normoglycaemia (Ikeya et al., 2002; Park et al., 2014). m-NSCs possess axon terminals in the larval endocrine gland and on the aorta, from which Dilps are released into the circulatory system. These insulin-producing cells (IPCs) function as *Drosophila* counterparts of mammalian pancreatic β-cells (Ikeya et al., 2002; Rulifson et al., 2002). In addition to the well-conserved insulin-like peptides and signaling pathways, the experimental procedures used to identify *Drosophila* IPCs and readily induce gene expression exclusively in the IPCs encouraged us to use this model organism. Using *Drosophila* as a model organism can enable investigation of the conserved mechanisms whereby IPCs become damaged, leading to IPC dysfunction and/or loss in diabetes. To establish T1D models in *Drosophila*, genetic ablation to remove only the IPCs has been performed by ectopic induction of proapoptotic gene *reaper* in the cells (Rulifson et al., 2002; Ikeya, et al., 2002; Broughton et al., 2005; Ueishi et al., 2009; Inoue et al., 2018). It resulted in a reduced viability, a delay in development and production of undersized adults. However, this genetic method is not natural and harsh for the organism; it was important to confirm whether the similar phenotypes appear in the organisms with accumulation of common types of cell stress like ER stress in the IPCs. *Drosophila* can serve as a useful model for studying connections between ER stress and diabetes.

Only two experimental systems that can induce ER stress are available in *Drosophila*. One of these systems was established when developing a *Drosophila* model of autosomal dominant retinitis pigmentosa, for which mutations in the rhodopsin gene were responsible. Ectopic expression of a mutant form of the *Drosophila* Rhodopsin-1 (Rh1) protein, *Rh1^G69D^*, resulted in the production of a misfolded rhodopsin variant that induced the UPR when it was expressed in the retina (Ryoo and Steller, 2007; Ryoo et al., 2007; Kang et al., 2012). Another stress model was previously established, wherein ectopic overexpression of the *Drosophila* presenilin (*Psn*) gene induces ER stress (Demay et al., 2014). Human Psn genes are thought to be responsible for familial Alzheimer's disease (Campion et al., 1995; Kovacs et al., 1996; Tu et al., 2006). Psn is a regulator of calcium flux in the ER, and its overexpression can modify calcium homeostasis in *Drosophila* and mammalian cells (Michno et al., 2009; Honarnejad et al., 2013). Psn overexpression in *Drosophila* wing imaginal discs induced ER stress, which activated the PERK/ATF4 branch of the UPR mediated by JNK signaling. Psn overexpression also resulted in caspase-dependent apoptosis (Demay et al., 2014). These previous studies indicated that chronic ER stress induces both JNK-dependent and JNK-independent apoptosis in *Drosophila*, suggesting that complex mechanisms of ER stress-induced apoptosis are conserved between *Drosophila* and mammals. However, the details of the pathway and factors involved in apoptosis are not well understood yet. Explicit evidence of ER stress-induced destruction or dysfunction of *Drosophila* IPCs has not been investigated yet. Therefore, establishing another *Drosophila* model with IPCs in a state of ER stress would be quite useful for investigating the relationship between ER stress and IPC destruction.

Thus, we focused on how ER stress causes the destruction of IPCs, resulting in the onset of diabetes, and tried to examine the mechanism at the individual level in *Drosophila*. In this study, we showed that expression of a dominant-negative form of Hsc70-3 (Hsc70-3^DN^), which is a *Drosophila* orthologue of ER chaperone, induced ER stress in *Drosophila* tissues. We further demonstrated that the Hsc70-3^DN^-induced ER stress model was useful for studying ER stress because it can induce greater stress than previous models and can be used to study various tissues. Using the new *Drosophila* model to study the pathogenesis of diabetes, we showed that ER stress-induced destruction of IPCs mediated by apoptosis was responsible for onset of the disease.

## RESULTS

### Ectopic expression of a dominant-negative form of ER chaperone (Hsc70-3^DN^) triggered the UPR in *Drosophila* tissues

**Fig. 1.**
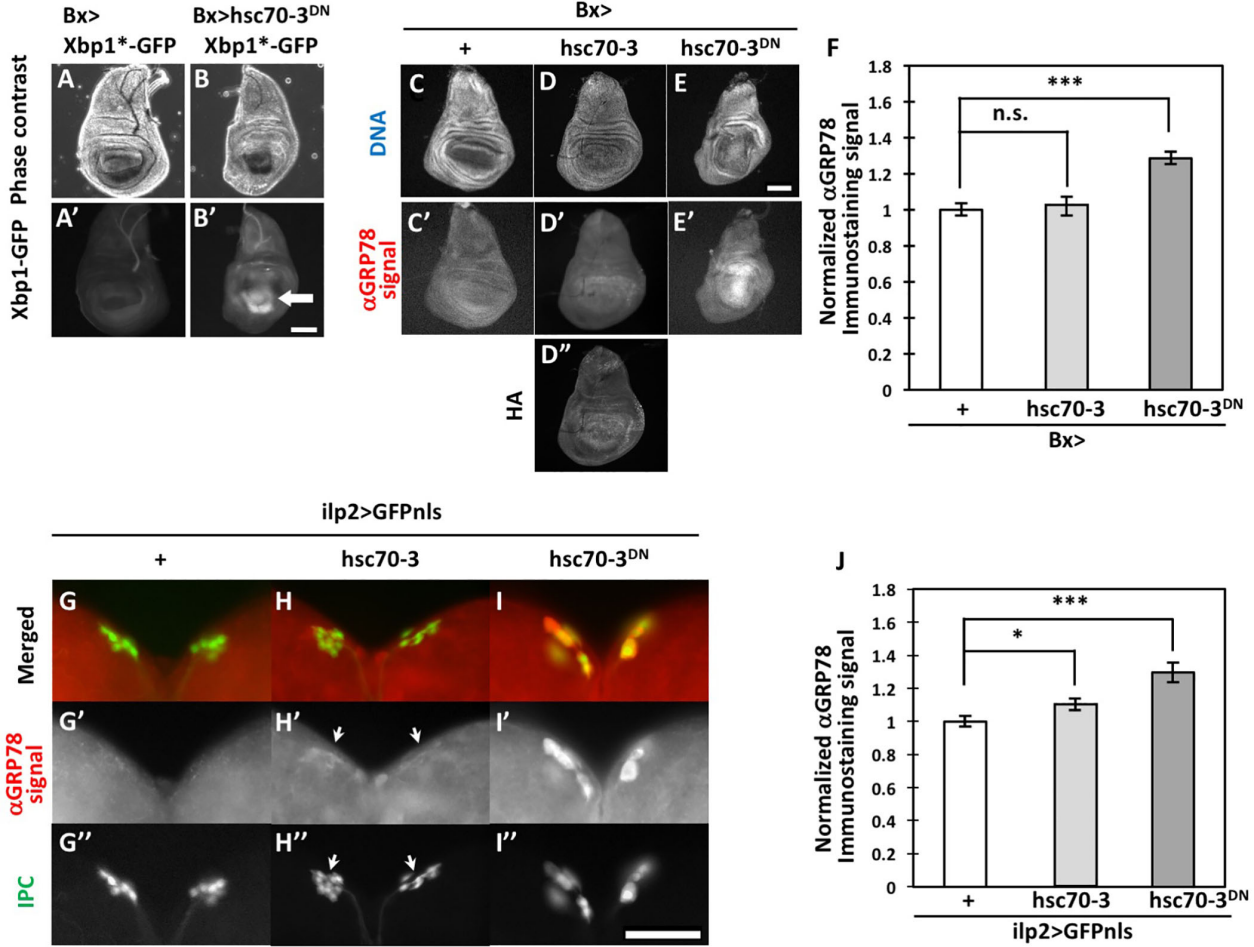
**Induction of UPR observed in wing imaginal discs and IPCs with ectopic Hsc70-3^DN^ expression.** (A–B) Expression of Xbp1-GFP generated by ER stress-dependent splicing of xbp1*-GFP mRNA in wing imaginal discs. Phase contrast (A,B) and fluorescence (A′,B′) micrographs of wing imaginal discs. (A,A′) Control wing disc (Bx>xbp1*-GFP). (B,B′) Wing disc expressing a dominant-negative form of Hsc70-3 in the wing pouch region (arrow) (Bx>hsc70-3^DN^, xbp1*-GFP). (C–E) Fluorescence micrograph of wing discs stained with DAPI (white). (C′–E′) Immunostaining of the wing discs with an anti-GRP78 antibody. (D″) Immunostaining of the wing disc with anti-HA antibody. (C,C′) Fluorescence micrograph of a control wing imaginal disc (*Bx-Gal4/+*). (D–D″) Wing imaginal disc expressing control Hsc70-3 in the wing pouch region of the imaginal disc (*Bx>hsc70-3*). (E,E′) Wing imaginal disc expressing a dominant-negative form of Hsc70-3 in the same region (*Bx>hsc70-3^DN^*). Anti-GRP78 immunostaining is shown in white. Note that more intense immunofluorescence was observed exclusively in areas expressing Hsc70-3^DN^, but not the control protein. (A–F) Relative intensity of anti-GRP78 immunostaining in wing imaginal discs. Immunofluorescence signal intensity in each wing imaginal disc with the control Hsc70-3 (*n*=31) or Hsc70-3^DN^ (*n*=25) expression was calculated and normalized to the control value, which was set as 1.0 (*Bx-Gal4/+*) (*n*=25; n.s., not significant, *P*>0.05; ***P<0.001, Student's *t*-tests). Error bars represent s.e.m. (G–I) Anti-GRP78 immunostaining of IPCs expressing GFPnls in brains from third-instar larvae. (G) Control IPCs (*ilp2>GFPnls*), (H) IPCs expressing the control Hsc70-3 (*ilp2>hsc70-3, GFPnls*), (I) IPCs expressing Hsc70-3^DN^ (*ilp2>hsc70-3^DN^, GFPnls*). Anti-GRP78 immunostaining is colored in red (G–I; white in G′–I′). Nuclei of IPCs visualized by GFPnls expression are colored green (G–I; white in G″–I″). Arrows in H′ and H″ indicate positions of IPC cells. Note that remarkably higher immunostaining signal was observed in IPCs expressing Hsc70-3^DN^, but not the control protein. (J) Relative intensities of anti-GRP78 immunostaining in larval IPCs. Immunofluorescence signal intensities in each IPC expressing Hsc70-3 (*n*=25) or Hsc70-3^DN^ (*n*=21) were calculated and normalized to the control value of 1.0 (*ilp2>GFPnls*) (*n*=21, *P<0.05, ***P<0.001, Student's *t*-test). Error bars represent s.e.m. Scale bars: (A–E) 100 µm, (G–I) 50 µm.

To achieve an efficient ER stress that triggers the UPR in *Drosophila* tissues, we induced targeted expression of a dominant-negative mutant of Hsc70-3, which is a *Drosophila* orthologue of the mammalian ER chaperone (Elefant and Palter, 1999) by *Drosophila* Gal4/UAS system. We found that it can activate the UPR. We first examined whether induction of Hsc70-3^DN^ could induce expression of an ER stress reporter, Xbp1*-GFP in wing imaginal discs (Fig. 1A,B). ER stress triggers splicing of *Xbp1* pre-mRNA (Xbp1*) such that it encodes a truncated Xbp1 precursor by removing an extra exon containing a stop codon. When the full-length Xbp1-GFP protein was synthesized from a mature *Xbp1* mRNA in response to ER stress, we observe GFP fluorescence after irradiation. In control (*Bx-Gal4/+*) wing imaginal discs, GFP fluorescence was not observed due to absence of ER stress-dependent splicing (Fig. 1A′). In contrast, intense GFP fluorescence was observed exclusively in the wing pouch region of wing imaginal discs expressing Hsc70-3^DN^ and Xbp1*-GFP mRNA simultaneously (Bx>hsc70-3^DN^, Xbp1*-GFP) (arrow in Fig. 1B′).

To confirm these results, we further investigated whether the expression of Hsc70-3^DN^ activated the UPR by examining expression of other targets. It was previously reported that *Drosophila* ER chaperone(s) and/or Hsp70 family proteins recognized by an anti-GRP78 antibody are upregulated under ER stress condition (Ham et al., 2014). We observed immunostaining signals in the imaginal discs expressing Psn, a known ER stress inducer, by ectopic expression, whereas a lower anti-GRP78 immunostaining signal at a basal level was observed in control wing discs (*Bx-Gal4/+*) (Fig. S1A′,C′,G). Thus, we next performed the same immunostaining of imaginal discs and IPCs expressing Hsc70-3^DN^. In control wing imaginal discs (*Bx-Gal4/+*), only the background signal was observed (Fig. 1C′–E′). Similarly, a less strong signal above background was observed in the discs expressing the control Hsc70-3 by *Bx-Gal4* (Fig. 1D′,F, *P*>0.05, *n*=25). We confirmed by anti-HA immunostaining that the protein was induced by the Gal4/UAS system (Fig. 1D″). In contrast to the control signal, the signal intensity significantly rose in the wing disc regions of the discs having ectopic expression of Hsc70-3^DN^ using the same Gal4 driver (Fig. 1D′,F, *P*<0.001, *n*=25). The raised immunostaining intensity suggests that expression of Hsp70 family proteins including ER chaperones increased considerably in response to ER stress by expression of the dominant-negative form of the ER chaperone, although we cannot identify the UPR-responsible protein(s). We further examined whether induction of Hsc70-3^DN^ could activate expression of another UPR target in wing imaginal discs (Fig. S2). It is known that ER stress triggers expression of the 4E-BP gene, a target of the PERK pathway (Kang et al., 2017). We examine whether expression of the gene can be induced by ectopic expression of Hsc70-3^DN^ using a *p{lacW}Thor^k13517^* enhancer trap line. In control (*Bx-Gal4/Y; Thor^k13517^/+*) wing imaginal discs and the discs expressing control Hsc70-3 (*Bx-Gal4/Y; Thor^k13517^/+; UAS-Hsc70-3/+*), a weak signal of anti-LacZ immunostaining was observed (Fig. S2A,B). In contrast, we observed slightly higher anti-LacZ immunostaining signal in wing imaginal discs expressing Hsc70-3^DN^ (*Bx>hsc70-3^DN^, Thor^k13517^/+*) (arrow in Fig. S2C′,C″). These data are consistent with the observations mentioned above. However, the difference between the control signals and the signal by Hsc70-3^DN^ expression was subtle. We evaluated that the enhancer trap line is not suitable for further experiments. Thus, we decided to examine expression of the UPR targets by anti-GRP78 immunostaining. Taken together with the observation that Hsc70-3^DN^ can induce ER stress-induced alternative splicing of *xbp1* mRNA, we conclude that ectopic expression of Hsc70-3^DN^ could induce ER stress that activates UPR-mediated pathways in *Drosophila* tissues.

Subsequently, we examined whether Hsc70-3^DN^ expression in IPCs using an IPC-specific Gal4 driver, *ilp2-Gal4*, similarly increased expression of the ER stress marker. We induced the expression of GFP proteins possessing a nuclear-localization sequence (NLS) exclusively in larval IPCs (Fig. 1G–I). As a result, anti-GRP78 immunostaining signal was significantly increased in IPCs expressing Hsc70-3^DN^ (Fig. 1I′,J, *P*<0.001, *n*=21), whereas the immunostaining signal was barely detected in control IPCs (*ilp2>GFPnls*) (Fig. 1G′, *n*=24). By contrast, a slight (10%) increase was detected in IPCs expressing the control Hsc70-3 (Fig. 1H′,J, *P*<0.05, *n*=25). These observations suggest that ectopic expression of Hsc70-3^DN^ could induce ER stress that activates an UPR in *Drosophila* tissues.

### Hsc70-3^DN^-induced ER stress resulted in apoptosis induction in IPCs

**Fig. 2.**
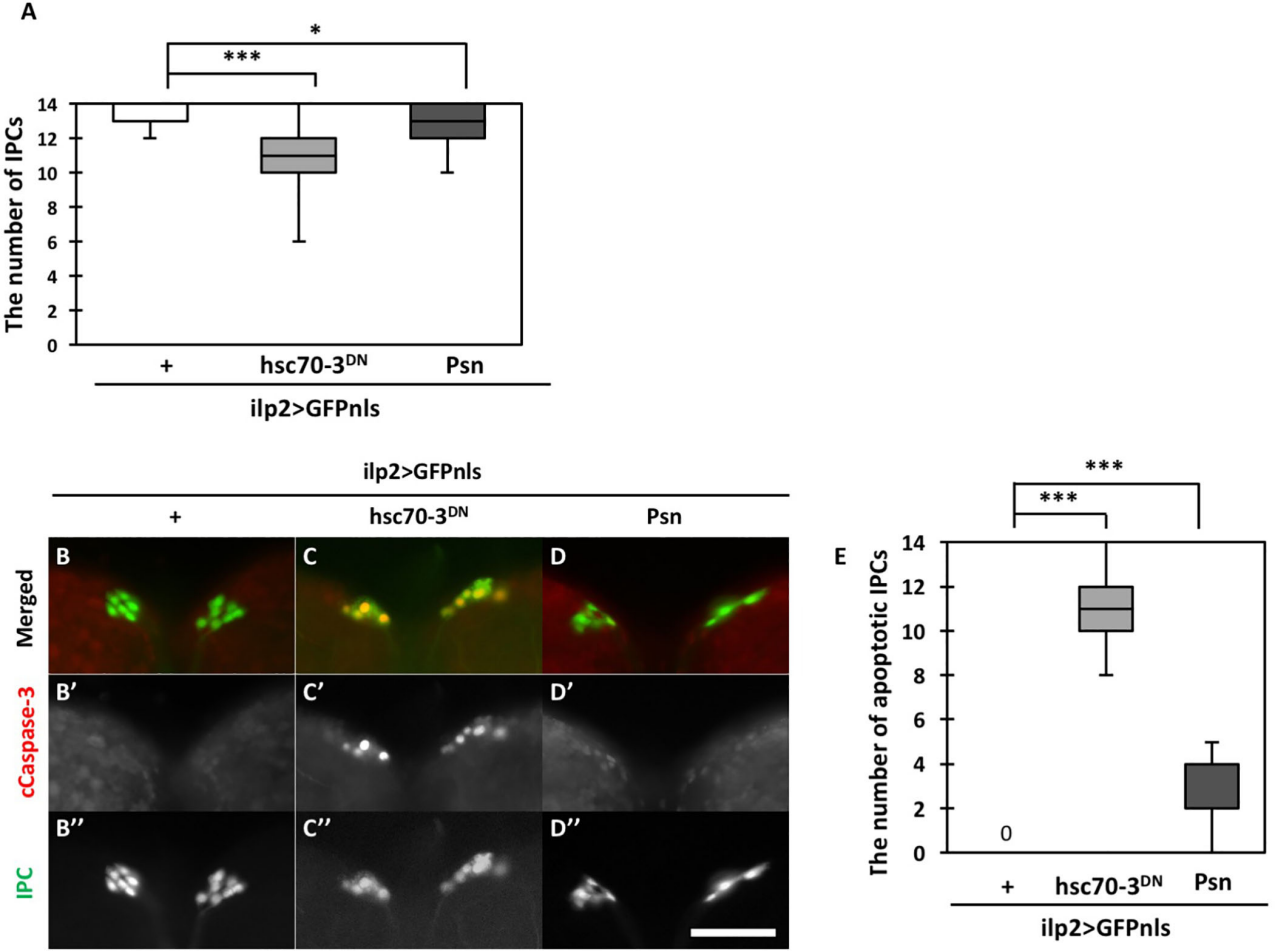
**Reduced numbers of IPCs and caspase activation in IPCs expressing either Hsc70-3^DN^ or Psn.** (A) Quantification of the numbers of larval IPCs in control third-instar larvae, larvae expressing Hsc70-3^DN^ and larvae expressing Psn. Note that the numbers of IPCs significantly decreased in both *ilp2>GFPnls, hsc70-3^DN^* larvae and *ilp2>GFPnls, Psn* larvae (*n*>31, *P<0.05, ***P<0.001, Mann–Whitney's *U*-test; error bars represent s.e.m.). (B–D″) Anti-CC3 immunostaining of larval IPCs expressing GFPnls. (B) IPCs of control larva (*ilp2>GFPnls*). (C) IPCs expressing Hsc70-3^DN^ (*ilp2>hsc70-3^DN^, GFPnls*). (D) IPCs expressing Psn (*ilp2>Psn, GFPnls*). Red, CC3 immunostaining signal; green: nuclei of IPCs. Scale bar: 50 μm. (E) Quantification of the numbers of larval apoptotic IPCs stained with anti-CC3 antibody. Note that the apoptosis in IPCs significantly increased in both *ilp2>GFPnls, hsc70-3^DN^* larvae and *ilp2>GFPnls, Psn* larvae compared with control larvae in which no apoptotic IPCs were observed (*n*>21, ***P<0.001, Mann–Whitney's *U*-test; error bars represent s.e.m.).

It has been speculated that IPC dysfunction and disruption eventually leads to the development of human T1D. Thus, we examined whether the accumulation of ER stress in IPCs could reduce the numbers of these cells. As a previous study reported that Psn overexpression in imaginal discs induces ER stress-induced apoptosis (Demay et al., 2014), we compared it with a loss of IPCs by ectopic expression of Hsc70-3^DN^ in larvae raised under continuous Psn expression in IPCs throughout development (Fig. S1D–F). We detected 13 IPCs on average in larval brains with an *ilp2>Psn, GFPnls* (*n*=46), whereas an average of 14 IPC cells was found in control brains (*ilp2>GFPnls*) (*n*=31) (Fig. S1H). The average number of IPCs also decreased in *ilp2>Psn, GFPnls* by 7.1%, compared with that in controls (Fig. 2A). In contrast, we counted 11 IPCs on average in *ilp2>hsc70-3^DN^, GFPnls* brains, decreasing by 21.4% compared with controls. (*n*=38). These data suggest that IPCs were subjected to considerably severe cellular damage by Hsc70-3^DN^-induced ER stress. Thus, we performed immunostaining with an anti-cleaved caspase-3 (CC3) antibody to determine whether apoptosis occurred in the larval IPCs (Fig. 2B–D). In control larval IPCs (*ilp2>GFPnls*), we observed very weak background staining (Fig. 2B′,E). In contrast, we found distinctive immunostaining signals in 92.7% of IPCs expressing Hsc70-3^DN^ (*n*=22) (Fig. 2C′,E), 18.2% of which expressed Psn (*n*=21) (Fig. 2D′,E). These results clearly indicate that the accumulation of ER stress promoted by either Hsc70-3^DN^ or Psn in IPCs induces caspase 3-dependent apoptosis, which results in loss of IPCs. Moreover, apoptosis induction by Hsc70-3^DN^ expression is more efficient than by Psn expression.

### ER stress-induced apoptosis was dependent on caspase-9-like caspase, Dronc, and the caspase-3-like caspases Drice and Dcp-1

**Fig. 3.**
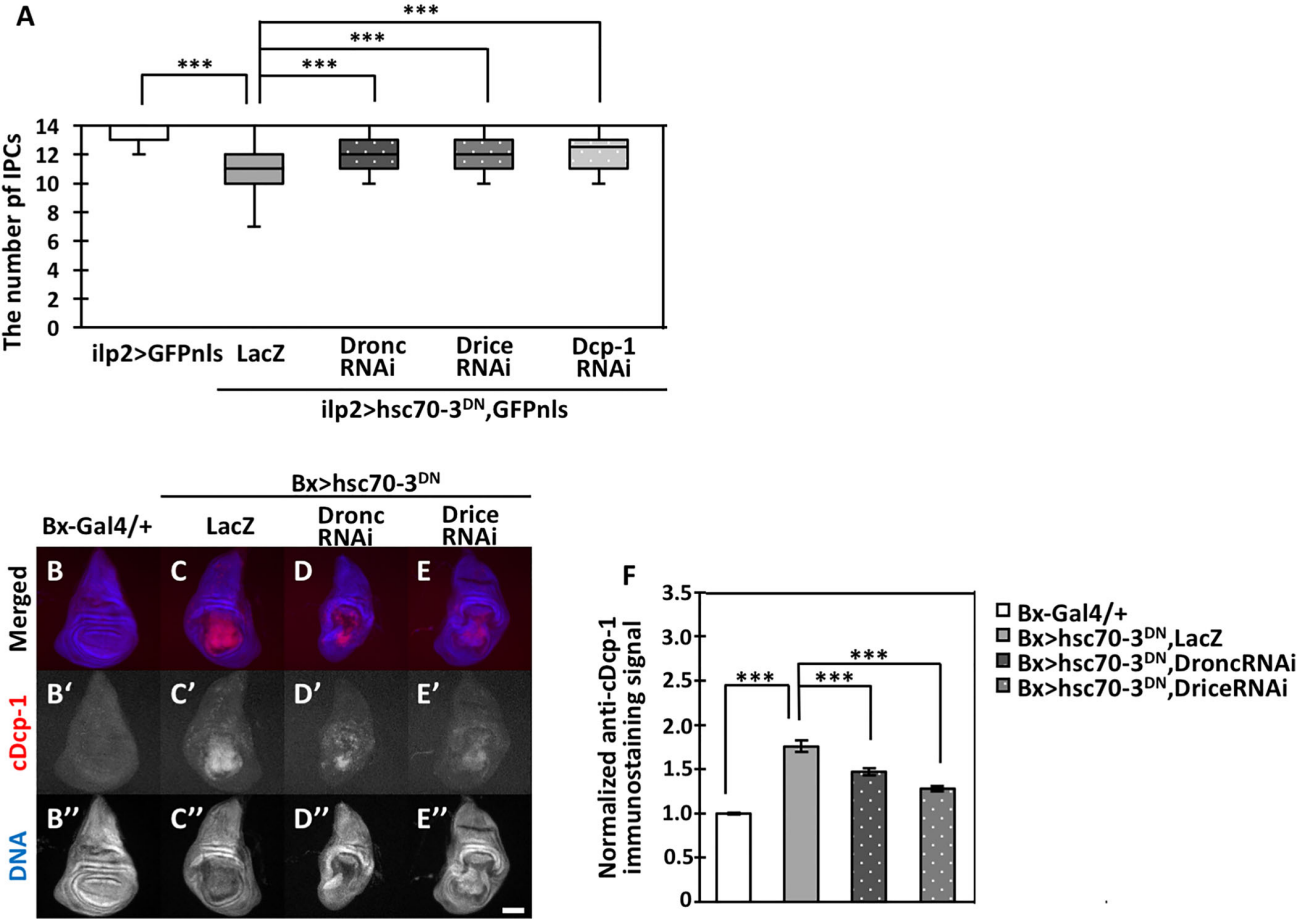
**Depletion of Dronc, Drice or Dcp-1 caused by expression of corresponding dsRNAs alleviated ER stress-induced apoptosis.** (A) Quantification of IPCs from third-instar larvae with simultaneous expression of Hsc70-3^DN^ and either lacZ mRNA or a dsRNA for the caspases, Dronc, Drice or Dcp-1. Note that the reduced number of IPCs due to ER stress accumulation was significantly rescued by depleting one of the three types of caspases (*n*>22, ***P<0.001, Mann–Whitney's *U*-test; error bars represent s.e.m.). (B–E″) Immunostaining of wing imaginal discs from third-instar larvae with an anti-cleaved Dcp-1 (cDcp-1) antibody. (B) Normal control wing disc (*Bx-Gal4/+*). (C) A wing disc expressing Hsc70-3^DN^ and LacZ simultaneously in the wing pouch region of the disc (*Bx>hsc70-3^DN^, LacZ*). (D) Wing imaginal disc expressing Hsc70-3^DN^ and dsRNA of *dronc* (*Bx>hsc70-3^DN^, DroncRNAi*). (E) Wing imaginal disc expressing Hsc70-3^DN^ and dsRNA of *drice* (*Bx>hsc70-3^DN^, DriceRNAi*). In B–E, anti-cDcp-1 immunostaining signal and DNA staining are colored in red (white in B′–E′) and blue (white in B″–E″), respectively. Scale bar: 100 µm. (F) Quantification of cDcp-1 signals in wing imaginal discs. The intensities of cDcp-1 signals in each wing imaginal disc with simultaneous expression of Hsc70-3^DN^ and either of LacZ mRNA or a dsRNA for one of each caspase were calculated and normalized to that of the control, set to 1.0 (*Bx-Gal4/+*) (*n*>12, ***P<0.001, Student's *t*-test; error bars represent s.e.m.).

To reveal the mechanism whereby the Hsc70-3^DN^-induced ER stress in IPCs causes apoptosis, we tried to identify caspases involved in the ER stress-induced apoptosis. Previously, it was reported that a *Drosophila* orthologue of caspase-9, Dronc, was involved in ER stress-induced apoptosis by Psn overexpression (Demay et al., 2014). To determine whether Dronc was required for Hsc70-3^DN^-induced apoptosis, we induced simultaneous expression of Hsc70-3^DN^ and a double-stranded RNA (dsRNA) against *dronc* mRNA. As a control for inducing Hsc70-3^DN^ without dsRNA, we generated individuals carrying *UAS-Hsc70-3^DN^* and *UAS-LacZ* to adjust the number of UAS sequences. Simultaneous expression of Hsc70-3^DN^ and LacZ in IPCs (*ilp2>hsc70-3^DN^, GFPnls, LacZ*) resulted in a significant reduction of IPCs (Fig. 3A). The average number of IPCs decreased by 21.4% compared to those in control *ilp2>GFPnls* flies (*P*<0.001, *n*=31) (Fig. 3A). The reduction in IPC number was significantly reversed by Dronc depletion in *ilp2>hsc70-3^DN^, GFPnls, Dronc RNAi* flies (*n*=30, *P*<0.001) (Fig. 3A).

To further confirm these results, we performed immunostaining experiments to detect caspase activation in wing imaginal discs rather than in IPCs because of an easier management of the immunostaining procedure (Fig. 3B–E). In wing discs simultaneously expressing Hsc70-3^DN^ and LacZ, we observed strong signals of anti-cleaved-Dcp-1 immunostaining (*n*=32, Fig. 3C′), whereas no signals over the background were observed in control wing discs (*Bx-Gal4/+, n*=23) (Fig. 3B′). These data indicate that Dcp1 can be activated in the discs after ER stress induction by Hsc70-3^DN^-induced expression. The immunofluorescence signal was significantly suppressed by the depletion of Dronc compared with the signal in *ilp2>hsc70-3^DN^, GFPnls, LacZ* (*P*<0.001, *n*=15, Fig. 3D′,F). These results suggest that Dronc was required for executing Hsc70-3^DN^-induced apoptosis. In addition, apoptosis required another caspase, caspase-3, which is activated by caspase-9 in many cases. In *Drosophila*, Drice and Dcp-1 have been reported as homologues of caspase-3. To examine whether these caspases are also required for Hsc70-3^DN^-induced apoptosis, we induced simultaneous expression of Hsc70-3^DN^ and dsRNA against *drice* mRNA. We used *UAS-drice RNAi* stock, which enables depletion of mRNAs for the respective caspases. Consistently, we observed a reduced anti-cDcp-1 immunostaining signal in the imaginal discs with the Drice depletion (*Bx>hsc70-3^DN^, DriceRNAi*) (Fig. 3E). This is consistent with the aforementioned result that reduction of IPCs following ER stress accumulation was also significantly rescued by Drice depletion (*ilp2>hsc70-3^DN^, GFPnls, DriceRNAi*, *P*<0.001, *n*=22, Fig. 3A). Although we demonstrated that Dcp1 is activated by the Hsc70-3^DN^-induced ER stress induction (Fig. 3C), we were unable to clarify whether Dcp1 is also involved in the ER stress-induced apoptosis by depletion of Dcp1 and immunostaining experiments with anti-c-DCP1 antibody. Therefore, these results suggest that at least Dronc and Drice were involved in ER-stress-induced apoptosis. Although off-targets of these RNAi stocks have not been reported, we would hope that the results would be confirmed by genetic analysis using null mutations of the caspase genes.

### Ectopic expression of Hsc70-3^DN^ in IPCs resulted in the appearance of diabetes-like growth inhibition phenotypes

**Fig. 4.**
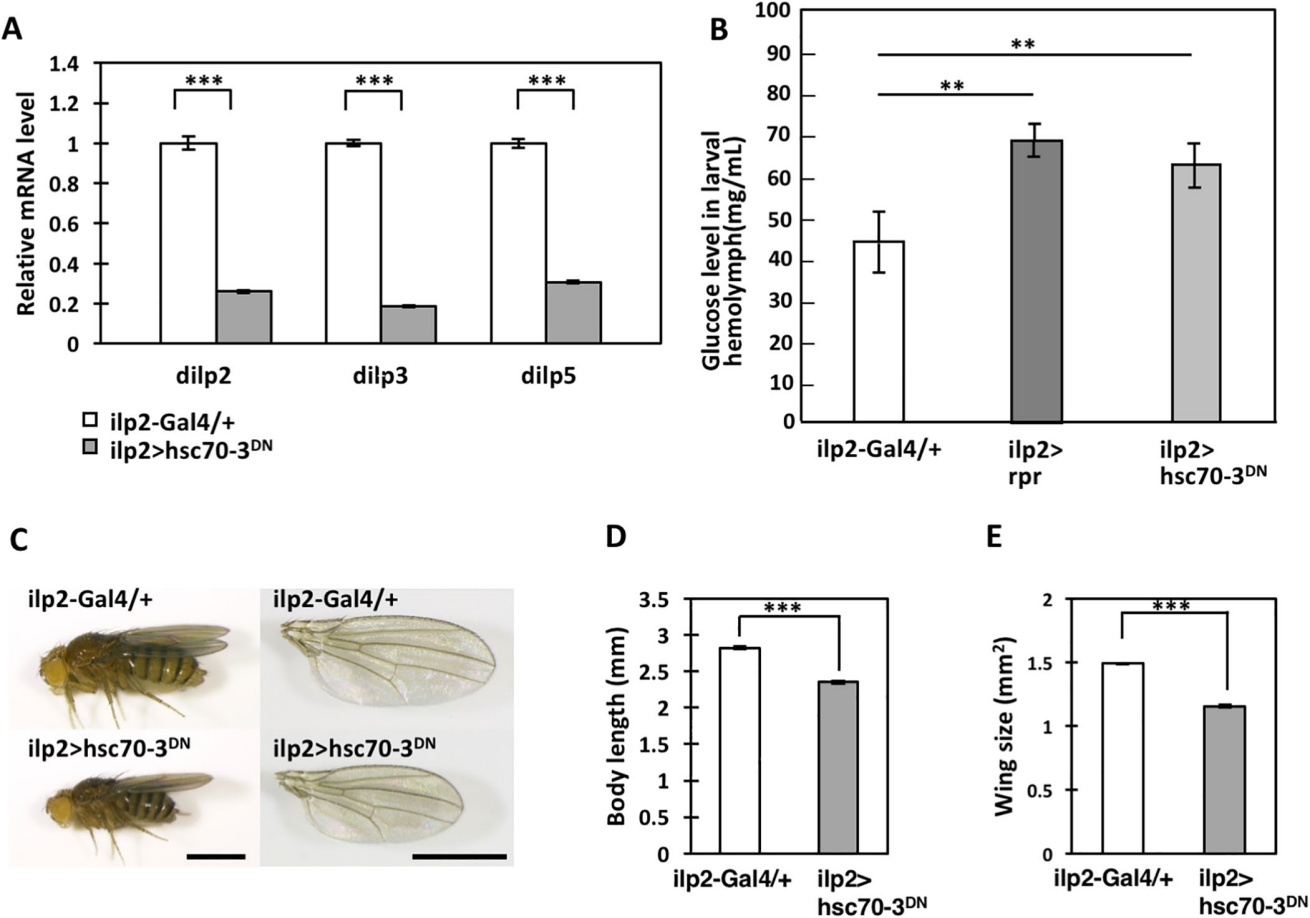
**Reduced expression of Dilps and growth-inhibition phenotypes observed in *Drosophila* expressing Hsc70-3^DN^ in larval IPCs.** (A) Relative mRNA-expression levels of *dilp2*, *3* and *5*, which encode *Drosophila* insulin-like peptides. Total RNA was prepared from adult heads in which the IPCs existed. White bars: relative levels of each *dilp* mRNA in control adult females (*ilp2-Gal4/+*). Grey bars: relative levels of each *dilp* mRNA in adult females raised under continuous Hsc70-3^DN^ expression in IPCs (*ilp2>hsc70-3^DN^*) throughout development. Relative levels were calculated and normalized to each mRNA level in control flies, which was set to 1.0 (***P<0.001, Student's *t*-test; error bars represent s.e.m.). (B) Glucose levels in the haemolymph from third-instar larvae. Control (*ilp2-Gal4/+*) glucose levels, those of larvae having ectopic expression of the pre-apoptotic gene *rpr* (*ilp2>hsc70-3^DN^*) and Hsc70-3^DN^ (*ilp2>rpr*) in IPCs. Ectopic expression of these genes in the cells elevated glucose levels in the larval hemolymph, compared with control [*n*≥3 (10 larvae per replicate), **P<0.01, Student's *t*-test; error bars represent s.e.m.]. (C–E) Adult phenotypes of female flies derived from individuals having targeted expression of Hsc70-3^DN^ in IPCs through the larval to adult stages. Note that Hsc70-3^DN^ expression in the cells resulted in the production of adults with a smaller body length with smaller wings compared with those in control flies (*ilp2-Gal4/+*). (C) Side-view images of female flies (left panels) and their wings (right panels). Upper panels indicate a control female fly as well as its wing (*ilp2-Gal4/+*). Lower panels indicate a female fly and its wing where the fly was raised under continuous expression of Hsc70-3^DN^ in the IPCs. Scale bars: 1 mm. (D) Quantification of adult body lengths (*n*>33, ***P<0.001, Student's *t*-test; error bars represent s.e.m.). (E) Quantification of adult wing sizes (*n*>58, ***P<0.001, Student's *t*-test; error bars represent s.e.m.).

It has been speculated that chronic ER stress in IPCs results in the development of diabetes in mammalian models (Papa, 2012). To test this hypothesis using a *Drosophila* model, we examined whether targeted expression of Hsc70-3^DN^ in *Drosophila* IPCs triggers diabetes-like phenotypes. As mentioned in the previous section, accumulation of Hsc70-3^DN^-induced ER stress in IPCs resulted in apoptosis. Thus, we next examined whether production of insulin-like peptides (Dilps) was inhibited. mRNA-expression levels of three Dilps (Dilp2, 3 and 5) were determined in IPCs in adult brains by performing qRT-PCR using total RNA prepared from adult heads. Compared with mRNA levels of these three genes in control adult females (*ilp2-Gal4/+*), the mRNA levels of Dilp2, 3 and 5 in *ilp2>hsc70-3^DN^* flies decreased by 74.0%, 81.5% and 69.3% of each control, respectively (Fig. 4A). Thus, we next examined whether ectopic expression of Hsc70-3^DN^ in the IPCs resulted in increased glucose level in their hemolymph. In third-instar larvae with ectopic expression of proapoptotic gene *reaper* in the IPCs (*ilp2>rpr*), the mean glucose level in their hemolymph was higher (69.5±3.1 mg/dl, *n*=3) (Fig. 4B) than that of the control (*ilp2-Gal4/+*) (45.1±7.2 mg/ml, *n*=6). Consistent with growth defects seen in *ilp2>hsc70-3^DN^*, we observed significantly higher glucose levels (62.4±4.1 mg/ml, *n*=3) in haemolymph collected from larvae expressing Hsc70-3^DN^ in their IPCs (*ilp2>hsc70-3^DN^*) (*P*<0.01) (Fig. 4B).

Next, we observed the phenotype of adults in which Hsc70-3^DN^ was continuously expressed in IPCs throughout development (*ilp2>hsc70-3^DN^*). It is known that induction of IPC-specific apoptosis results in similar growth defects including hyperglycemia in larval hemolymph, a developmental delay and growth retardation at larval stage, and finally undersized adult flies emerged (Rulifson, et al., 2002; Ikeya, et al., 2002; Broughton et al., 2005; Inoue et al., 2018). Adults raised under this condition showed smaller whole bodies with smaller wings compared with controls (*ilp2-Gal4/+*) (Fig. 4C–E). The average body length of female flies raised under this condition was 83.5% of that of control flies. Similarly, the whole-wing size of *ilp2>hsc70-3^DN^* adult females was calculated to be 77.4% of controls. As mentioned in the previous section, accumulation of Hsc70-3^DN^-induced ER stress in IPCs resulted in apoptosis. Taken together, these data suggest that accumulation of ER stress by continuous expression of the dominant-negative mutant of a *Drosophila* ER chaperone in IPCs resulted in the production of diabetes-like phenotypes.

### Hsc70-3^DN^-induced ER stress can produce similar, but more severe phenotypes than Psn-induced ER stress

**Fig. 5.**
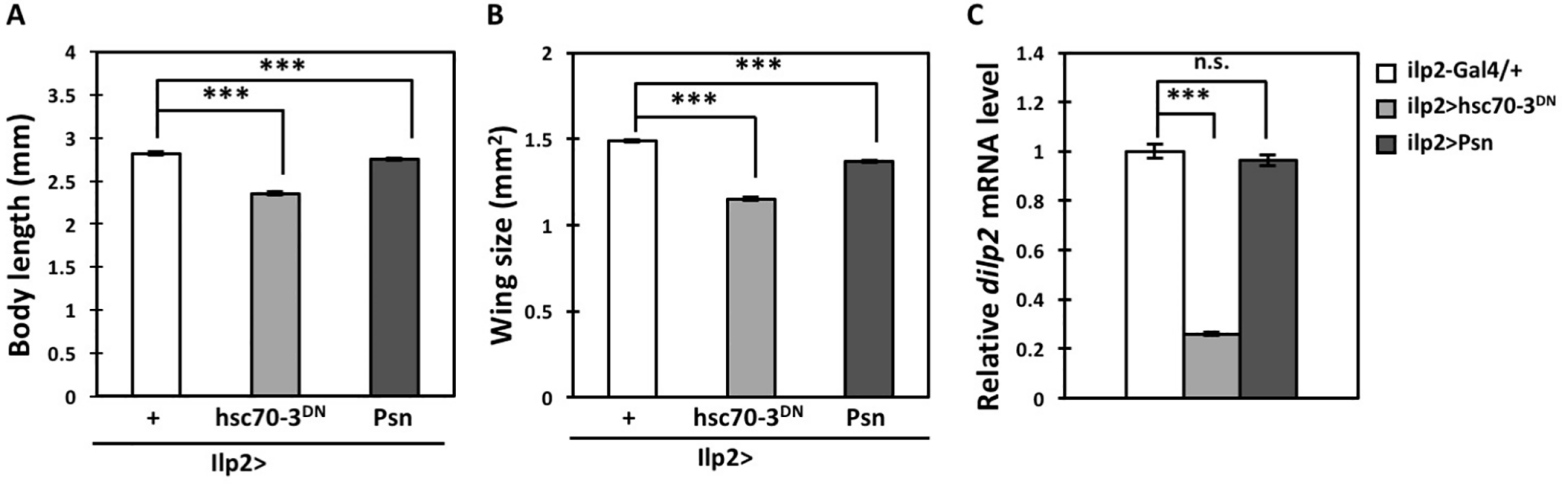
**Growth inhibition phenotypes and reduced expression of Dilps observed in *Drosophila* expressing Psn and that expressing Hsc70-3^DN^ in larval IPCs.** (A) Quantification of adult body lengths (*n*>33, ***P<0.001, Student's *t*-test; error bars represent s.e.m.). (B) Quantification of adult wing sizes (*n*>58, ***P<0.001, Student's *t*-test; error bars represent s.e.m.). (C) Relative *dilp2* mRNA-expression levels. Total RNA was prepared from heads of control (*ilp2-Gal4/+*) adult females, expressing Hsc70-3^DN^ in IPCs (*ilp2>hsc70-3^DN^*), or Psn in IPCs (*ilp2>Psn*). *Dilp2* mRNA levels in adult females raised under simultaneous Hsc70-3^DN^ or Psn expression in IPCs throughout development were calculated and normalized to those of controls, which were set to 1.0 (*ilp2-Gal4/+*) (n.s., not significant, ***P<0.001, Student's *t*-test; error bars represent s.e.m.).

To confirm the finding that the accumulation of ER stress in IPCs triggered diabetes-like phenotypes, we induced the ER stress according to another previously published protocol (Demay et al., 2014). Overexpression of Psn, which is known as a gene responsible for Alzheimer's disease, triggered the UPR in *Drosophila* wing imaginal discs (Demay et al., 2014). We induced Psn expression in wing imaginal discs under *Bx-Gal4* (*Bx>Psn*) or in IPCs throughout development (*ilp2>Psn*) and examined whether Hsp70 family proteins including the ER chaperone(s) was induced by anti-GRP78 immunostaining (Fig. S1A′–C′,G). The immunostaining signal was considerably induced in wing discs with *Bx-Gal4*-induced ectopic expression of Psn (Fig. S1C′), whereas a much lower signal was seen in the same region of control imaginal discs (*Bx-Gal4/+*) (Fig. S1A′). Similarly, Psn overexpression also raised the immunostaining signal in IPCs (Fig. S1D′–F′,H). The signal induced by Psn expression appeared less intense in both wing imaginal discs and IPCs. The differences in immunofluorescence intensity between control cells and cells overexpressing Psn was not statistically significant (Fig. S1H). It is difficult to compare with the signal intensity observed after expressing Hsc70-3^DN^ by anti-GRP78 immunostaining, as the antibody can recognize Hsc70-3 protein expressing dependent on the Gal4 driver as well as endogenous ER chaperone(s) induced as a consequence of the UPR (Fig. S1B′,C′,E′,F′,G,H). Even so, the effects of Psn overexpression in IPCs on the growth-inhibition phenotypes appeared less severe compared with those in adult females raised under continuous Hsc70-3^DN^ expression (Fig. 5A,B). It was consistent with the results of the immunostaining data described above. The average body length of flies with continuous Psn expression in IPCs was 97.7% of that of the controls. Similarly, the whole-wing size of Psn-expressing adult females was 92.0% of the controls. Furthermore, the level of *dilp2* mRNA in adult brains expressing Psn within IPCs also decreased by 3.6% compared with the controls (Fig. 5C). However, this decline was less pronounced compared with the 70% decrease seen in adults raised under continuous Hsc70-3^DN^ expression in IPCs through development. These observations support the conclusion that ER stress accumulating in IPCs triggered similar diabetes-like phenotypes, such as growth inhibition (Rulifson et al., 2002; Inoue et al., 2018). Moreover, our findings suggest that expression of Hsc70-3^DN^ generated more severe and more distinctive responses, resulting in the production of ER stress-induced phenotypes in *Drosophila* tissues compared with a known Psn-induction protocol.

### Reduced *dilp2* mRNA expression and growth-inhibition phenotype generated by ER stress in IPCs was prevented by depletion of the Dronc caspase

If it was possible to inhibit ER stress-induced apoptosis in IPCs, then the diabetes-like phenotypes resulting from a loss of IPCs could be prevented. Therapeutic agents and remedies for preventing apoptosis could serve as targets for anti-diabetes treatments. Therefore, we investigated whether the depletion of caspases in IPCs could restore the reduced amount of *dilp2* mRNA and eventually suppress ER stress-induced growth inhibition. Adult females with ER stress accumulation in IPCs were created by the continuous expression of Hsc70-3^DN^ (*ilp2>hsc70-3^DN^, LacZ*), which resulted in the production of significantly smaller flies with smaller wings. The average body length of *ilp2>hsc70-3^DN^, LacZ* female flies was 2.28 mm, which was 81.0% of the length of controls (*ilp2-Gal4/+*) (Fig. S3A). The average wing area of the female flies was 1.15 mm^2^, which was 77.4% of the area in control flies (*ilp2-Gal4/+*) (Fig. S3B). We found that the reduction of body length by ER stress in IPCs was significantly rescued by the depletion of Dronc (*ilp2>hsc70-3^DN^, DroncRNAi*) (*P*<0.001, *n*=30) (Fig. S3A). Consistent with this result, Drice depletion also significantly rescued the reduction of body length caused by ER stress (*ilp2>hsc70-3^DN^, DriceRNAi*) (*P*<0.001, *n*=34) (Fig. S3A). Subsequently, we simultaneously depleted Dcp-1, which rescued the reduction of body length caused by ER stress (*ilp2>hsc70-3^DN^, Dcp-1RNAi*) (*P*<0.001, *n*=34) (Fig. S3A). However, in contrast with the results of the adult body length, the reduction in wing size by ER stress in IPCs was not significantly rescued by the depletion of each caspase in IPCs (Fig. S3B). Furthermore, we examined whether inhibiting IPC apoptosis by depleting those caspases could restore the lowered levels of *dilp2* mRNA in adult female brains, as described above (Fig. S3C). The mRNA levels of *dilp2* in adult females with ER stress-accumulation in IPCs decreased to 18% of the control (*ilp2-Gal4/+*). In contrast, the *dilp2* mRNA levels in adults with ER stress accumulation and *dronc* depletion in IPCs increased by 15% (Fig. S3C). In contrast with the results for Dronc depletion, the *dilp2* mRNA levels in females with simultaneous depletion of Drice in IPCs was not significantly different (*P*=0.25). These results allowed us to conclude that the reduction of *dilp2* mRNA expression induced by ER stress accumulation in IPCs was significantly suppressed by the depletion of Dronc caspases, although the difference was less remarkable. These results are consistent with the suppression effect observed after caspase depletion on the growth-inhibition phenotype that appeared in adult bodies and were derived from ER-stress induced apoptosis in IPCs.

### Hsc70-3^DN^ expression activated the JNK pathway in the wing disc cells and its activation was required for subsequent apoptosis

**Fig. 6.**
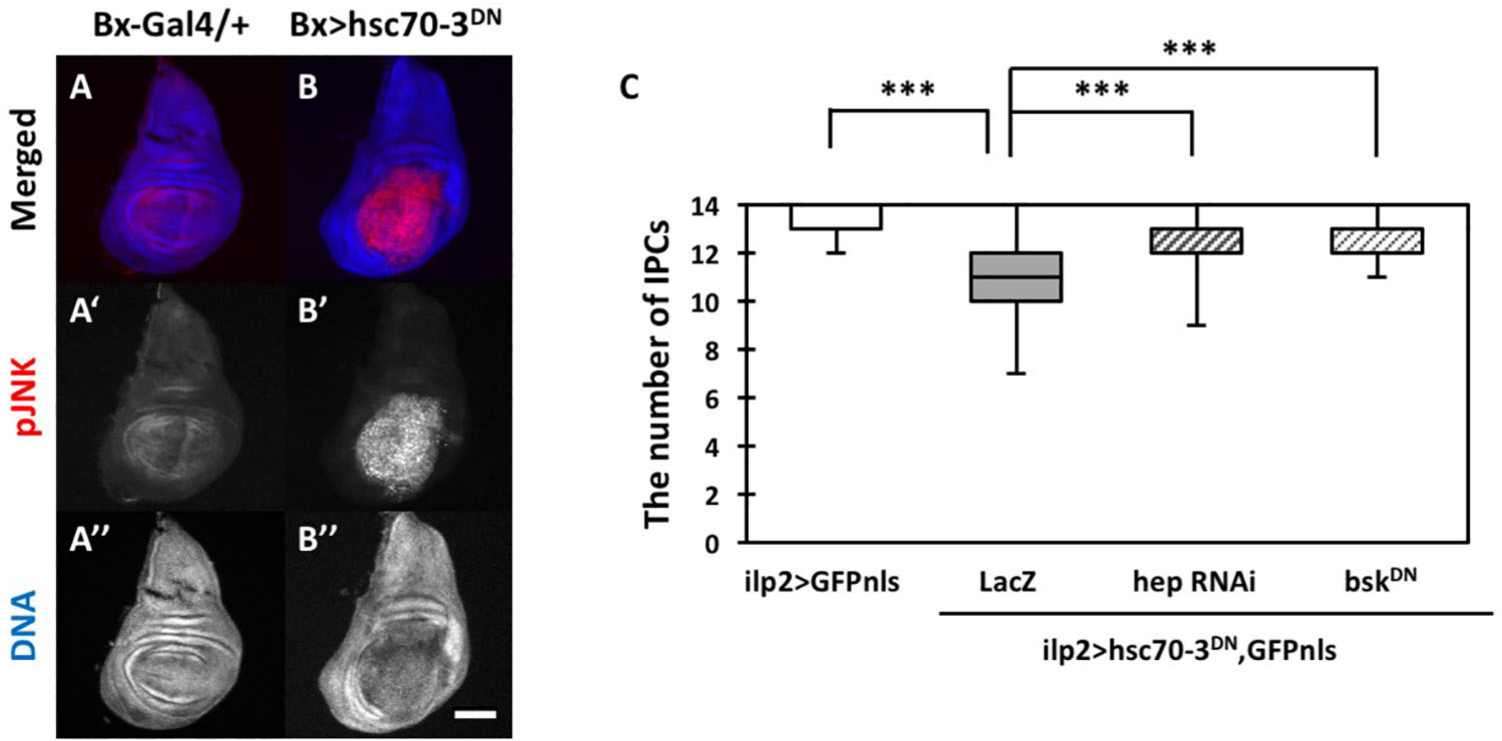
**Immunostaining of wing discs expressing Hsc70-3^DN^ with an antibody against activated JNK and the rescue of reduced IPC numbers by simultaneous depletion of JNK-signaling factors.** (A–B″) Immunostaining of wing imaginal discs with accumulated ER stress from third-instar larvae. (A–A'') Control (*Bx-Gal4/+*) wing disc. (B–B'') Wing discs expressing Hsc70-3^DN^ in the wing porch area, depending on *Bx-Gal4*. In A and B, anti-pJNK immunostaining signal and DNA staining are colored in red (white in A′–B′) and blue (white in A″–B″), respectively. Scale bar: 100 µm. (C) Quantification of IPCs in the brains from third-instar larvae. Note that the reduction of IPC numbers due to ER stress accumulation was significantly rescued by the depletion of *hep* (*ilp2>hsc70-3^DN^, hepRNAi, GFPnls*; *P*<0.001) or by expression of a dominant-negative form of Bsk (*ilp2>hsc70-3^DN^, bsk^DN^, GFPnls*; *n*>31, ***P<0.001, Mann–Whitney's *U*-test; error bars represent s.e.m.).

JNK is widely known as a key factor required for transducing stress signals and apoptosis occurring as a response to stress signaling. In a previous study using *Drosophila*, it was reported that ER stress can induce both JNK-dependent and JNK-independent apoptosis (Demay et al., 2014). To reveal the pathway whereby ER stress induced apoptosis in IPCs, we examined whether JNK was activated in response to ER stress accumulation. First, we observed JNK phosphorylation in *Drosophila* wing discs expressing Hsc70-3^DN^ by anti-pJNK immunostaining. In control (*Bx-Gal4/+*) wing discs, a weak striped pattern of pJNK immunostaining signal was observed (Fig. 6A′). It is known that the JNK activation in this region occurs during the normal development of *Drosophila* wing discs. However, a much more intense anti-pJNK signal was observed in wing disc regions expressing Hsc70-3^DN^ (Fig. 6B′). This finding indicates that ER stress induced by Hsc70-3^DN^ activated JNK signaling in *Drosophila* tissues. Next, we examined whether JNK signaling was required for the ER stress-induced apoptosis. To test this possibility, we induced continuous expression of both Hsc70-3^DN^ and dsRNA against JNKK (Jun kinase kinase) orthologue in IPCs. The orthologue is encoded by a *Drosophila hep* gene. The UAS-*hep* RNAi stock enables efficient depletion of *hep* mRNA. As stated above, the numbers of IPCs accumulating during ER stress (*ilp2>hsc70-3^DN^, GFPnls, LacZ*) decreased by 21.4%, compared with controls (*ilp2>GFPnls*). This reduction was significantly rescued by simultaneous depletion of *hep* (*ilp2>hsc70-3^DN^, GFPnls, hep RNAi*) (*P*<0.001, *n*=37) (Fig. 6C). To confirm this result, we further induced a simultaneous expression of dominant-negative form of Bsk, which is a *Drosophila* JNK, instead of dsRNA against *hep* mRNA. Consistently, the reduction of IPCs induced by ER stress accumulation was significantly rescued by downregulation of Bsk (*ilp2>hsc70-3^DN^, GFPnls, bsk^DN^*) (*P*<0.001, *n*=38) (Fig. 6C). These results suggested that the accumulation of ER stress induced in wing imaginal discs and IPCs induced apoptosis in a JNK-signaling-dependent manner.

### Inhibition of JNK signaling was insufficient for recovery of ER stress-induced growth phenotypes or reduced *dilp2* mRNA level in IPCs

We showed that apoptosis inhibition by caspase depletion, which resulted in the recovery of IPC reduction, partially rescued the reduced levels of *dilp2* mRNA and growth inhibition (Fig. S4). We also showed that the inhibition of JNK signaling, which is involved in inducing apoptosis, rescued the reduction of IPCs in Fig. 6C. Thus, we examined whether inhibition JNK signaling was sufficient for suppressing the phenotypes induced by ER stress in IPCs. As a consequence of ER stress induced by Hsc70-3^DN^ expression in IPCs throughout development (*ilp2>hsc70-3^DN^, LacZ*), undersized adults with smaller wings were generated. (Fig. S4A,B). We inhibited JNK signaling in IPCs by cell-specific depletion of *hep* mRNA in IPCs throughout development. We observed that the growth inhibition in adult females (*ilp2>hsc70-3^DN^, LacZ*) was significantly suppressed in *ilp2>hsc70-3^DN^, hepRNAi* adult females (*P*<0.001, *n*=31). We further investigated the effects of inhibiting signaling via cell-specific expression of Bsk^DN^. Consistent with the suppressive effect of *hep* RNAi on the growth phenotype (evaluated by measuring body lengths), the Bsk^DN^ expression suppressed the growth-inhibition phenotypes, as determined by measuring the body lengths of adults (*P*<0.001, *n*=33). In contrast to these results, however, the average wing area of adult female *ilp2>hsc70-3^DN^, hep RNAi* flies was not significantly larger than that of adult female *ilp2>hsc70-3^DN^, LacZ* flies. (Fig. S4B, *P*=0.14, *n*=44). Consistently, expressing Bsk^DN^ exclusively in IPCs (*ilp2>hsc70-3^DN^, bsk^DN^*) also did not significantly suppress the growth phenotype (Fig. S4B, *P*=0.074, *n*=53).

As we have obtained inconsistent results in terms of suppression of the growth phenotype by measuring either body lengths or wing areas, we next examined the expression level of *dilp2* mRNA in the brains of adult female flies. Accumulation of ER stress in IPCs (*ilp2>hsc70-3^DN^, LacZ*) resulted in reduced *dilp2* mRNA expression, the level of which was 18% of that in control flies (*ilp2-Gal4/+*). The *dilp2* mRNA level in brains with IPCs simultaneously expressing Hsc70-3^DN^ and dsRNA against *hep* mRNA (*ilp2>hsc70-3^DN^, hep RNAi*) was 18.4% of that in control flies (Fig. S4C). These results allowed us to conclude that inhibiting JNK signaling by depleting *hep* did not alter the reduced *dilp2* mRNA levels (*P*=0.33). Therefore, we conclude that the inhibition of JNK signaling by depleting its signaling factors was insufficient for recovery from ER stress-induced growth inhibition or reduced *dilp2* mRNA levels in IPCs.

## DISCUSSION

### Ectopic expression of a dominant-negative form of an ER chaperone (Hsc70-3^DN^) can induce strong ER stress in various tissues in *Drosophila*

It has been argued that the dysfunction of cells caused by ER stress accumulation or ER stress-induced apoptosis are responsible for development of some diseases, including neurodegenerative disease, diabetes, cancer, atherosclerosis and liver disease (Kaufman, 2002; Malhi and Kaufman, 2011; Zeeshan et al., 2016). Therefore, it is beneficial to establish an experimental system that can induce ER stress readily in various tissues, in order to study the relationships between ER stress and diseases. In *Drosophila*, two experimental procedures that can induce ER stress-mediated apoptosis are known. One procedure involves expressing a mutant of Rhodopsin (*Rh1^G69D^*). Another involves Psn overexpression (Ryoo et al., 2007; Kang et al., 2012; Demay et al., 2014). These systems have been used to induce ER stress in several *Drosophila* tissues, such as the compound eye, the wing and the accessory gland, which is a secretory tissue in the testes (Ryoo and Steller*,* 2007; Demay et al., 2014; Chow et al., 2015). However, these systems have not been applied for studying ER stress induction in IPCs. Neither of the previous systems appears to be adequate for that purpose because they can induce ectopic expression of neuron-related factors in non-neuronal cells. In fact, ectopic Psn expression in IPCs did not provide remarkable effects in IPCs. In contrast, our ER stress model, which involves Gal4-dependent ectopic Hsc70-3^DN^ expression, can be applied to IPCs. This model was useful in that it enabled us to induce ER stress more conveniently in various tissues by induction of the ubiquitously expressed protein. Using this system, we could test the hypothesis that ER stress triggers the ER stress-mediated cell damage, which results in tissue failure. Because ER stress induction by ectopic expression of Hsc70-3^DN^ offers several advantages over the previous systems in terms of its more efficient stress induction and availability in various tissues, Hsc70-3^DN^ can serve a powerful ER stress model with other tissues.

### Excess accumulation of ER stress in IPCs results in the appearance of diabetes-like phenotypes or symptoms

It has been argued that ER stress is related to the destruction or dysfunction of pancreatic β-cells in T1D (Oyadomari et al., 2002; Tersey et al., 2012; Engin et al., 2013; Lombardi and Tomer, 2017). However, it has not been established whether accumulated ER stress or reduced UPR is directly responsible for cell damage or cell death occurring in IPCs, which leads to diabetic development. To confirm this point, we induced ER stress exclusively in IPCs and observed both a loss of IPCs and a reduced expression of Dilps. We observed ER stress-triggered phenotypes reminiscent of diabetes phenotypes. Similar phenotypes also occurred in flies with continuous Psn expression in IPCs. These results support the hypothesis that ER stress itself is a major cause of cell damage in IPCs. In the well-characterized T1D murine model of the non-obese diabetic mouse, ER stress markers, such as Xbp1, GRP78 and CHOP, were induced or upregulated (Tersey et al., 2012; Marhfour et al., 2012). Another report showed that ER itself also resulted in misfolded morphology in the islets of NOD mice (Tersey et al., 2012). Moreover, in a T1D model mouse at the pre-diabetes stage, expression of UPR mediators, such as ATF6 and Xbp1, were downregulated. A treatment with tauroursodeoxycholic acid, which is known to alleviate ER stress, significantly reduced the incidence of diabetes and raised insulin secretion (Engin et al., 2013). These results support a hypothesis that ER stress is a key factor responsible for the disruption of IPCs in patients with diabetes.

Our ER stress-induced T1D model in *Drosophila* has some advantages over the NOD mice. The autoimmune T1D mouse model has a number of limitations in its use for the T1D study. For example, the cumulative diabetes incident varies among different institutes (Pozzilli et al., 1993; Chaparro and DiLorenzo, 2010). It is a polygenic model for autoimmune T1D. The innate immune system in NOD mice can influence the development of T1D, as the mice are genetically deficient in MyD88, an essential factor acting downstream of Toll-like receptor. The NOD mice have been shown to produce a lot of false-positive therapies (Reed and Herold, 2015). By comparison, our *Drosophila* model is responsible for extra ER stress accumulation by ectopic expression of Hsc70-3^DN^ in the IPCs. It may provide a potentially suitable alternative that would enable studies on basic aspects of intervention therapies of the disease. T1D studies using the *Drosophila* model cost much less compared with those using NOD mice. A high mortality of the mouse model, which sometimes troubles researchers, should be also solved.

We propose that our ER stress model can also be used for studying T2D. ER stress and UPR are involved in the development of T2D as well as T1D. The insulin resistance and hyperglycaemia associated with T2D are accommodated by increased proinsulin translation. Under these conditions, the UPR is activated to compensate for the increased protein-folding requirement in the ER. Prolonged activation of the UPR contributes to the β-cell death, leading to insulin resistance (Kaufman, 2002). In T2D models in mice or *Drosophila*, animal models fed a high-fat diet or high-sugar diet are commonly established (Rovira-Llopis et al., 2014; Sabio et al., 2008; Musselman et al., 2011). In *Drosophila*, the Gal4/Gal80 system is a further powerful tool for expressing a large amount of protein in restricted cells with a specified timing. Using this genetic tool, it is possible to induce ER stress in IPCs specifically at the adult stage to study the effects on aging. This capability enables the development of another promising disease model, mimicking the T2D condition.

### Hsc70-3^DN^-induced ER stress causes apoptosis through activating stress-responsive JNK signaling in IPCs

We demonstrated that Hsc70-3^DN^-induced ER stress can induce activation of the JNK-signaling pathway and apoptosis in IPCs. JNK is well known as a key factor for transducing stress signals and for inducing apoptosis occurring as a response to stress signaling. However, we failed to rescue the Hsc70-3^DN^-induced growth-inhibition phenotype by downregulation of the JNK pathway. As we stated previously, two *Drosophila* models are available for studying ER stress induction. ER stress induced both by ectopic expression of a rhodopsin mutant (*Rh1^G69D^*) and by ectopic Psn expression triggered apoptosis through a signaling pathway mediated by CDK5, MEKK1 and JNK (Ryoo et al., 2007; Kang et al., 2012). CDK5 is an atypical cyclin-dependent kinase functioning in differentiated post-mitotic cells, such as neurons, and pancreatic β-islet cells (Tsai et al., 1994; Connell-Crowley et al., 2000; Wei et al., 2005; Choi et al., 2010; Kang et al., 2012). As *Drosophila* IPCs are neurosecretory cells, it is likely that CDK5 also functions in *Drosophila* IPCs. In mouse insulinoma (MIN6) cells, a specific inhibitor of CDK5 raised insulin secretion. Furthermore, the inhibitor also increased insulin secretion in a T2D mouse model (Kitani et al., 2007). Thus, it is worthwhile to examine whether CDK5 is involved in apoptosis induction in IPCs subjected to continuous accumulation of ER stress through JNK signaling using this *Drosophila* diabetes model. In terms of treatments for diabetes, identifying factors that mediate apoptosis are important, because they are potentially novel targets for anti-diabetes therapies.

In conclusion, the major findings in our study are as follows. Ectopic expression of Hsc70-3^DN^ enabled development of a novel powerful model in which ER stress could be more efficiently induced in various *Drosophila* tissues. The accumulation of ER stress in IPCs triggered the onset and development of diabetes. ER stress activated JNK signaling and induced apoptosis in IPCs in a manner that was dependent on Dronc, Drice and Dcp-1.

## MATERIALS AND METHODS

### *Drosophila* stocks and husbandry

All *D.* *melanogaster* stocks were maintained on standard cornmeal food at 25°C, as previously described (Oka et al., 2015). Food: 7.2 g agar, 100 g glucose, 40 g dried yeast and 40 g of cornmeal were added into 1 l water, mixed and boiled while stirring constantly. After the food media had cooled down below 65°C, 5 ml of 10% parahydroxybenzonate dissolved in ethanol and 5 ml of propionic acid were added as antiseptics. Gal4-dependent expression was done at 28°C. *w^1118^* from Bloomington *Drosophila* Stock Center (BDSC; Bloomington, Indiana, USA) was used as a normal control stock. The following UAS stocks were used in this study, obtained from BDSC: *UAS-hsc70-3^D231S^* (*UAS-hsc70-3^DN^*) (#5841) (Elefant and Palter, 1999) and *UAS-GFPnls* (#4776). *UAS-Hsc70-3xHA* (*UAS-Hsc70-3*) (#108461) for induced expression of a normal Hsc70-3 was obtained from FlyORF (University of Zurich, Zurich, Switzerland). The following *UAS* stocks were obtained from Kyoto Stock Center (Kyoto, Japan): *UAS-LacZ* (#107532) and *UAS-bsk^DN^* (#108773). *UAS-LacZ* was used as a control of Gal4-dependent UAS expression. The following *UAS-RNAi* stocks were obtained from BDSC: *UAS-Dcp1RNAi* [*TripHMS01779*] (#38315), *UAS-DroncRNAi* (#23033) and *UAS-DriceRNAi* (#28064) were distributed by Vienna *Drosophila* Resource Center (VDRC, Vienna, Austria). All three *UAS-RNAi* stocks were confirmed to be capable of depleting each mRNA efficiently. Another *UAS-RNAi* stock, *UAS-hep RNAi* (#4353R-2), was obtained from the National Institute of Genetics (Mishima, Sihzuoka, Japan). The *UAS-hep RNAi* stock used allowed efficient depletion of endogenous *hep* mRNA (Neisch et al., 2010). *P{UAS-Xbp1.EGFP.LG}* was a gift from Prof. H. Steller (The Rockefeller University, USA) (Ryoo et al., 2007). The Gal4 driver stocks used in this study were as follows: *P{ilp2-GAL4.R}* (*ilp2-Gal4*) (BDRC, #37516) for IPC-specific expression and *P{GawB}Bx^MS1096-KE^* (*Bx-Gal4*) (BDRC, #8860) for wing pouch and marginal region-specific expression in wing discs. For monitoring *Thor* gene expression, we used *p{lacW}Thor^k13517^* (#9558) stock from BDSC.

### Xbp1-GFP reporter assay in response to ER stress

To detect the UPR signaling induced by ER stress accumulation in wing imaginal discs, we observed expression of Xbp1-GFP generated as a consequence of ER stress-induced splicing of the mRNA transcribed from *UAS-Xbp1.EGFP.LG.* Wing imaginal discs were dissected from matured third-instar larvae in 0.7% NaCl. The living discs were observed without fixation and observed under a conventional fluorescent microscope (IX81, Olympus, Tokyo, Japan,).

### Immunostaining procedures

Third-instar larvae were dissected in 0.7% NaCl to collect larval brains and wing discs. The tissue samples were fixed in 3.7% formaldehyde for 15 min at room temperature, consequently permeabilized in 0.1% PBST and blocked with 10% normal goat serum. To stain wing discs with anti-Caspase-3 antibody, 0.2% PBST was used instead of 0.1% PBST. The following primary antibodies were used at the dilution described; rabbit anti-β-galactosidase (MP Biomedicals, #55976) at 1:1000, rabbit anti-GRP78 (Bip) (StressMarq Biosciences Inc., Cadboro Bay, Victoria, Canada) that could recognize Hsp70 family proteins including Hsc70-3 in *Drosophila* at 1:500, rabbit Cleaved Caspase-3 (Asp175) (#9661, Cell Signaling, Danvers, Massachusetts, USA) at 1:200 for larval brain immunostaining and at 1:150 for wing disc immunostaining, rabbit anti-Cleaved *Drosophila* Dcp-1 (Asp216) (Cell Signaling, antibody #9578) at 1:500, and rabbit anti-phospho-SAPK/JNK (pThr183, pTyr185) (Calbiochem, La Jolla, CA, USA) at 1:200. This antibody was used to detect phosphor Bsk/JNK in *Drosophila*. Secondary antibody conjugated to Alexa Fluor 594 was purchased from Molecular Probes (Eugen, OR, USA) and used at 1:400. Samples were mounted in Vectashield (Vector Laboratories, Burlingame, CA, USA). DAPI (Sigma-Aldrich) was used at 1:1000 dilution to label the nuclei. Samples were observed with a fluorescent microscope (Olympus, Tokyo, Japan, model: IX81). Image acquisition was controlled through the Metamorph software version 7.6 (Molecular Devices) and processed with ImageJ or Adobe Photoshop CS. Immunofluorescence intensities in the wing pouch region of the wing discs, where Bx-Gal4-dependent gene expression occurred, were quantified using ImageJ software, and the intensity values were calculated and normalized to the background immunofluorescence intensities, measured in a wing disc region outside the wing pouch, that was set as 1.0. Comparisons of the two groups were performed using the Student's *t*-test. Statistical analyses were performed using GraphPad 10 Prism 6 (Select Science, Waltham, USA). Data were considered significant at *P*<0.05.

### Counting IPCs

To determine the number of IPCs, IPC nuclei were labelled by IPC-specific expression of GFPnls and the number of GFP-positive nuclei were counted in a set of whole-brain hemisphere. Brains of adults or third-instar larvae were dissected in 0.7% NaCl. The samples were fixed in 3.7% formaldehyde for 15 min at room temperature and subsequently mounted in Vectashield (Vector Laboratories, Burlingame, CA, USA). The samples were observed with a fluorescent microscope (Olympus, Tokyo, Japan, model: IX81). For IPC counting, at least 20 larvae of each genotype were used. Results are presented as bar graphs created using GraphPad Prism 6. Each dataset was assessed using Mann–Whitney's *U*-test. An *F*-test was performed to determine equal or unequal variance. When the *P*-value was less than 0.05, it was calculated using Mann–Whitney's *U*-test of unequal variance.

### Measurement of adult body length and wing area

Images of adult whole bodies and gross area of unprocessed wings were digitally captured using a Nikon Digital Sight camera. Measurement of adult body length from the anterior end of the head to the posterior end of the abdomen was performed using the manual measurement system of Nikon Digital Sight. Wing area of each image was measured using ImageJ software (NIH). Comparisons of the two groups were performed using the Student's *t*-test. Statistical analyses were performed using GraphPad 10 Prism 6 (Select Science). Data were considered significant at *P*<0.05.

### qRT-PCR analysis

Total RNA was extracted from heads of adult female flies using the Trizol reagent (Invitrogen) as described (Ueda et al., 2018). cDNA synthesis from total RNA was carried out using the Primer Script^®^ High Fidelity RT-PCR kit (TaKaRa, Shiga, Japan) including an oligo dT primer. Real-time qPCR was performed using the FastStart Essential DNA Green Master (Roche, Mannheim, Germany) on a Light Cycler Nano instrument (Roche, Mannheim, Germany). qPCR primers used in this study were as follows:

RP49-F, 5′-TTCCTGGTGCACAACGTG-3′,

RP49-R, 5′-TCTCCTTGCGCTTCTTGG-3′,

Dilp2-F, 5′-AGCAAGCCTTTGTCCTTCATCTC-3′,

Dilp2-R, 5′-ACACCATACTCAGCACCTCGTTG-3′,

Dilp3-F, 5′-ATGGCTTCGAAGACCGTTCCC-3′,

Dilp3-Rv, 5′-TCCATGGTGCACGACTTCAGG-3′,

Dilp5-F, 5′-GACTCCTGTTGCCGCAAATCG-3′ and

Dilp5-R, 5′-GATGGACCAGGTATCGCAGCA-3′.

Each sample was duplicated on the PCR plate, and the final results average three biological replicates. For the quantification, the ΔΔCt method was used to determine the differences between target gene expression relative to the reference *Rp49* gene expression.

### Glucose measurements

For a measurement of glucose concentration in larval haemolymph, ten mature third-instar larvae raised at 25°C were collected. Haemolymph from of ten larvae from each genotype was used for the glucose assay. Haemolymph was diluted (1:10) in homogenization buffer [137 mM NaCl, 2.7 mM KCl, 5 mM Tris (pH 6.6)] and heated for 5 min at 70°C. Subsequently, trehalose in the supernatant was converted into glucose by incubation with a porcine kidney trehalase (Sigma-Aldrich) at 37°C for overnight. Total glucose was measured using the glucose assay kit (#T8778, Sigma-Aldrich) for 15 min at 30°C as described (Rulifson et al., 2002). Quantifications were performed using a SmartSpec spectrophotometer (Bio-Rad) at 340 nm. We repeated the assay more than three times (ten larvae per replicate) and calculated the mean glucose level (mg per ml of hemolymph) in larvae with each genotype.

## Supplementary Material

Supplementary information
