# Supplementary material for: Endoplasmic reticulum stress-induced cellular dysfunction and cell death in insulin-producing cells results in diabetes-like phenotypes in Drosophila
Source: Biol Open. 2019 Dec 20;8(12):bio046524. doi: 10.1242/bio.046524 (PMC6955230; doi:10.1242/bio.046524)
Supplement: Supplementary information [file biolopen-8-046524-s1.pdf]

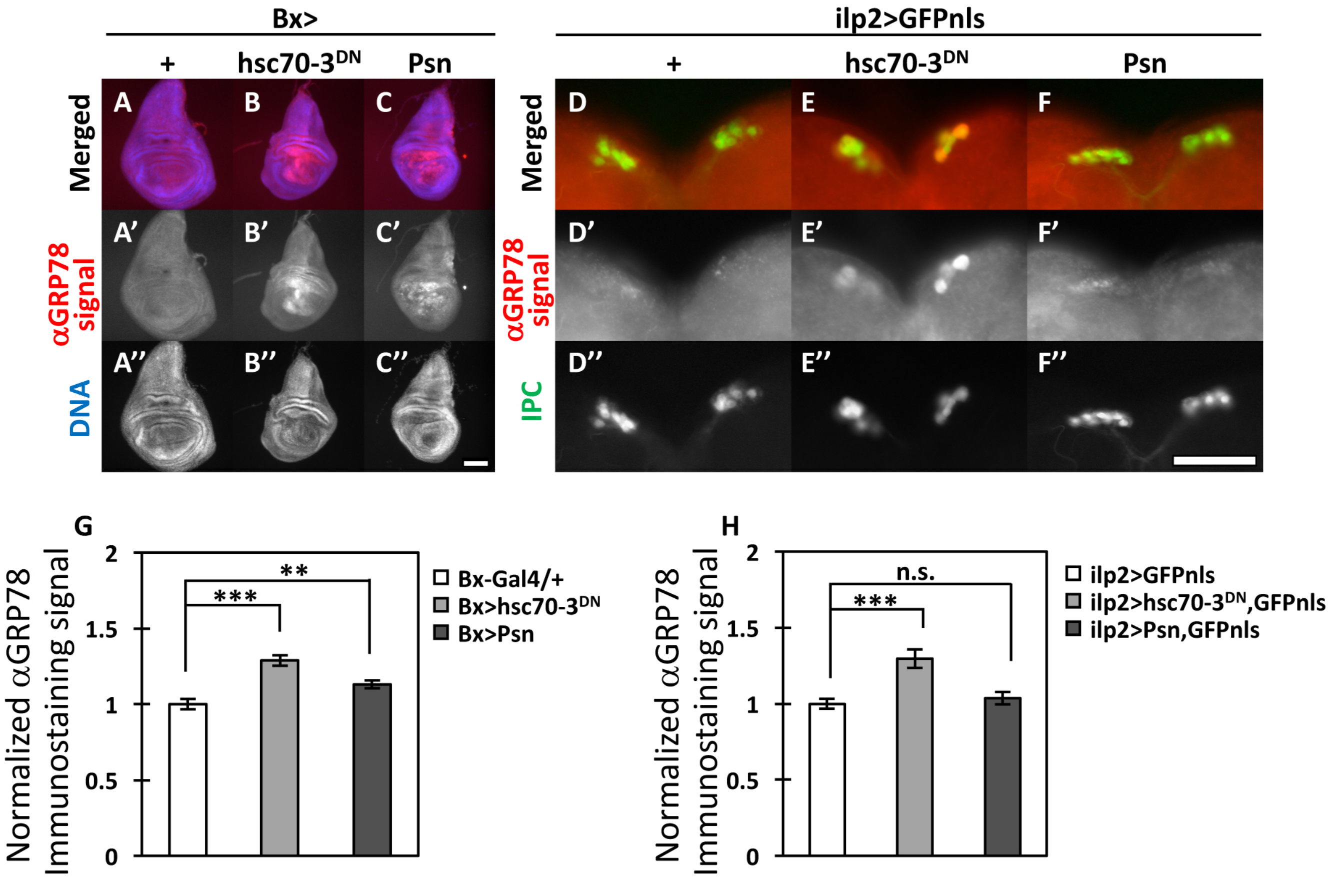

Fig. S1. Phenotypes of ER stress model expressing Psn and that expressing Hsc70-3^{DN} in wing discs and IPCs during development. (A–C) Anti-GRP78 immunostaining of wing imaginal discs. (A) A fluorescence micrograph of a control wing imaginal disc (*Bx-Gal4/+*, n = 26). (B) A wing imaginal disc expressing a dominant-negative form of Hsc70-3 in the wing pouch region (*Bx>hsc70-3^{DN}*). (C) A wing imaginal disc expressing Psn in the same region (*Bx>Psn*). In A–C, anti-GRP78 immunostaining signal and DNA staining are coloured in red (white in A'–C') and blue (white in A"–C"), respectively. Note that more intense GRP78 expression was exclusively observed in the area expressing Hsc70-3^{DN} compared to areas expressing Psn. Scale bar, 100 µm. (D–F) Anti-GRP78 immunostaining of IPCs expressing GFPnls in larval brains from third-instar larvae. (D)

Biology Open • Supplementary information

Control IPCs (*ilp2>GFPnls*, n = 35). (E) IPCs expressing Hsc70-3^{DN} (*ilp2>hsc70-3^{DN}*, *GFPnls*). (F) IPCs expressing Psn (*ilp2>Psn, GFPnls*). Anti-GRP78 immunostaining signal is coloured in red (D–F, white in D'–F'). Nuclear GFPnls expression in IPCs is coloured in green (D–F, white in D''–F''). Note that remarkably higher anti-GRP78 immunostaining signal was observed in IPCs expressing Hsc70-3^{DN}, compared with IPCs expressing Psn. Scale bar, 50 μm. (G) Relative intensities of GRP78 signals in wing imaginal discs. The intensity of GRP78 signals in each wing imaginal disc with either Hsc70-3^{DN} or Psn expression was calculated and normalized to that of the control, which was set to 1.0 (*Bx-Gal4/+*) (n > 13, $**p < 0.01$, $***p < 0.001$, Student's t-test). The error bars represent SEMs. (H) Quantification of GRP78 signals of larval IPCs. The intensities of GRP78 signals in each wing imaginal disc expressing Hsc70-3^{DN} or Psn were calculated and normalized to that of the control, which was set to 1.0 (*ilp2>GFPnls*) (n > 18, n.s.; not significant, $p > 0.05$, $***p < 0.001$, Student's *t*-test). The error bars represent SEMs.

Biology Open • Supplementary information

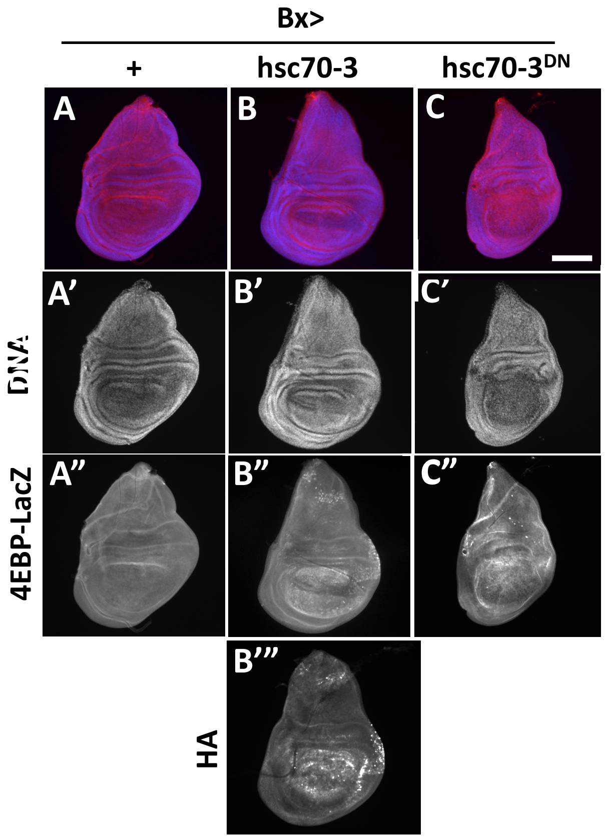

Fig. S2. Anti-LacZ immunostaining to examine *4E-BP* gene expression in wing imaginal discs having ectopic expression of Hsc70-3^{DN} using an enhancer trap line of *thr* gene. (A–C) Anti-LacZ immunostaining of wing imaginal discs prepared from 3rd instar larvae. (A) A fluorescence micrograph of a control wing imaginal disc (*Bx-Gal4/+*). (B) A wing imaginal disc expressing control Hsc70-3 (Hsc70-3HA) in the wing pouch region (*Bx>hsc70-3*). (C) A wing imaginal disc expressing Hsc70-3^{DN} in the wing pouch region (*Bx>hsc70-3^{DN}*). In A–C, DNA staining and anti-LacZ immunostaining signal are coloured in red (white in A'–C') and blue (white in A"–C"), respectively. (B"') Anti-HA immunostaining to confirm expression of the Hsc70-3HA. Scale bar, 100 µm.

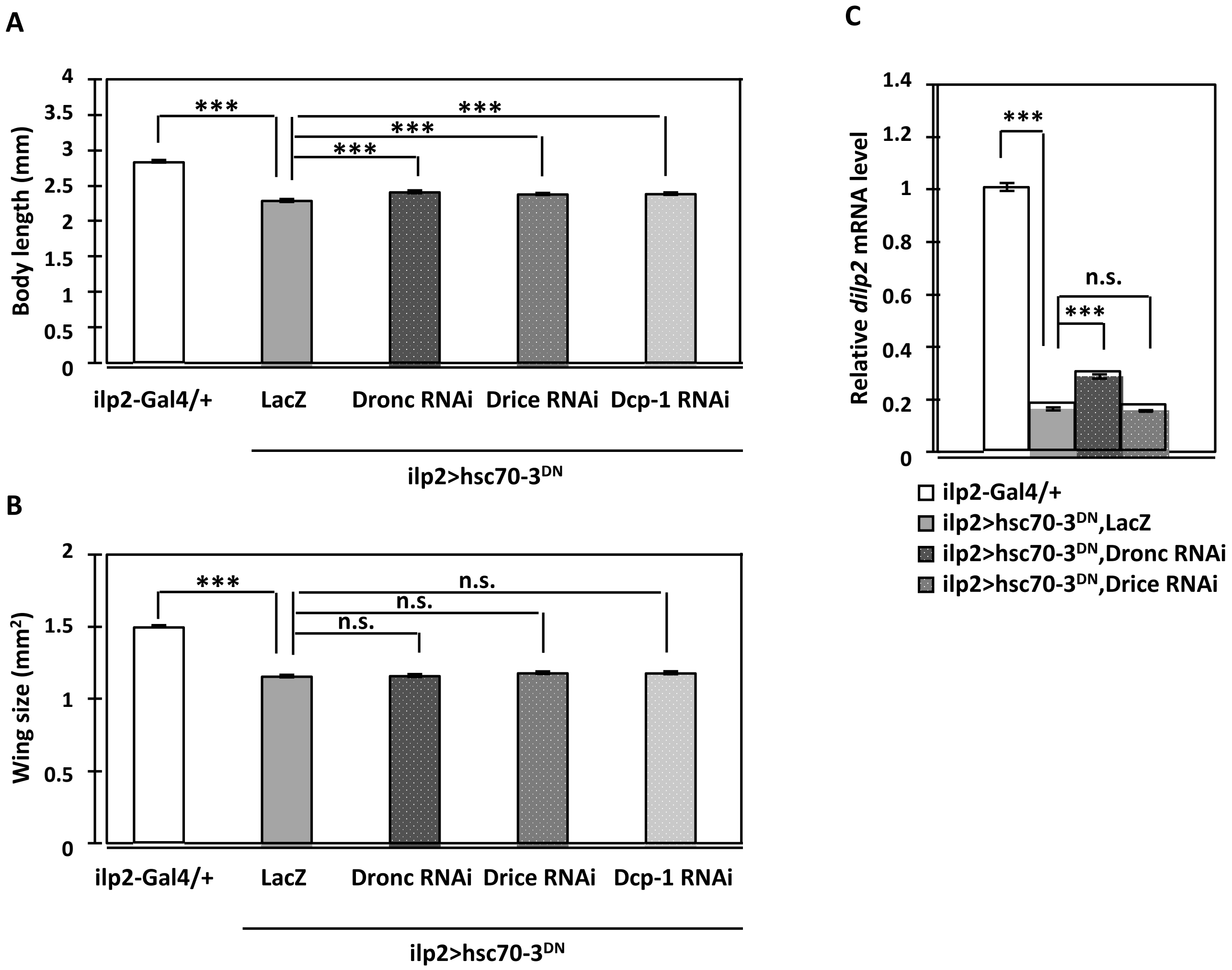

Fig. S3. Effects of caspase depletion by dsRNA expression on IPC survival, *dilp2* mRNA levels, and the ER stress induced-growth phenotype. (A) Quantification of adult body lengths of adult females raised under simultaneous expression of Hsc70-3^{DN} and dsRNA for each caspase in IPCs throughout development. The body lengths of adults expressing Hsc70-3^{DN} in IPCs were significantly shorter than those of controls (*ilp2-Gal4/+*). The shortened lengths of adults with Hsc70-3^{DN} expression in the IPCs was significantly suppressed in adults with simultaneous expression of Hsc70-3^{DN} and

Biology Open • Supplementary information

depletion of Dronc, Drice, or Dcp-1 (n > 30, ***$p < 0.001$, Student's *t*-test). Error bars represent SEMs. (B) Quantification of adult wing sizes. The wing sizes of adults expressing Hsc70-3^{DN} in IPCs were significantly smaller than those of controls (*ilp2-Gal4/+*). The reduced wing sizes of adults with Hsc70-3^{DN} expression in the IPCs were significantly suppressed in adults with simultaneous expression of Hsc70-3^{DN} and depletion of Dcp-1 (n > 37, ***$p < 0.001$, Student's *t*-test), whereas the wing areas were not significantly changed by simultaneous depletion of Dronc, Drice, and Dcp-1 (n.s.; not significant ($p > 0.05$). Error bars represent SEMs. (C) Quantification of the mRNA levels of *dilp2*. Total RNA was prepared from adult heads of control (*ilp2-Gal4/+*) females, those with ER stress in IPCs (*ilp2>hsc70-3^{DN}, LacZ*), those with expression of Hsc70-3^{DN} and depletion of *dronc* in IPCs (*ilp2>hsc70-3^{DN}, DroncRNAi*), those with expression of Hsc70-3^{DN} and depletion of *drice* in IPCs (*ilp2>hsc70-3^{DN}, DriceRNAi*), and those with expressing of Hsc70-3^{DN} and depletion of *dcp-1* in IPCs (*ilp2>hsc70-3^{DN}, Dcp-1RNAi*). Relative *dilp2* mRNA-expression levels in each adult female were calculated and normalized to the control level, which was set to 1.0 (*ilp2-Gal4/+*) (n.s.; not significant ($p > 0.05$, ***$p < 0.001$, Student's *t*-test).

Biology Open • Supplementary information

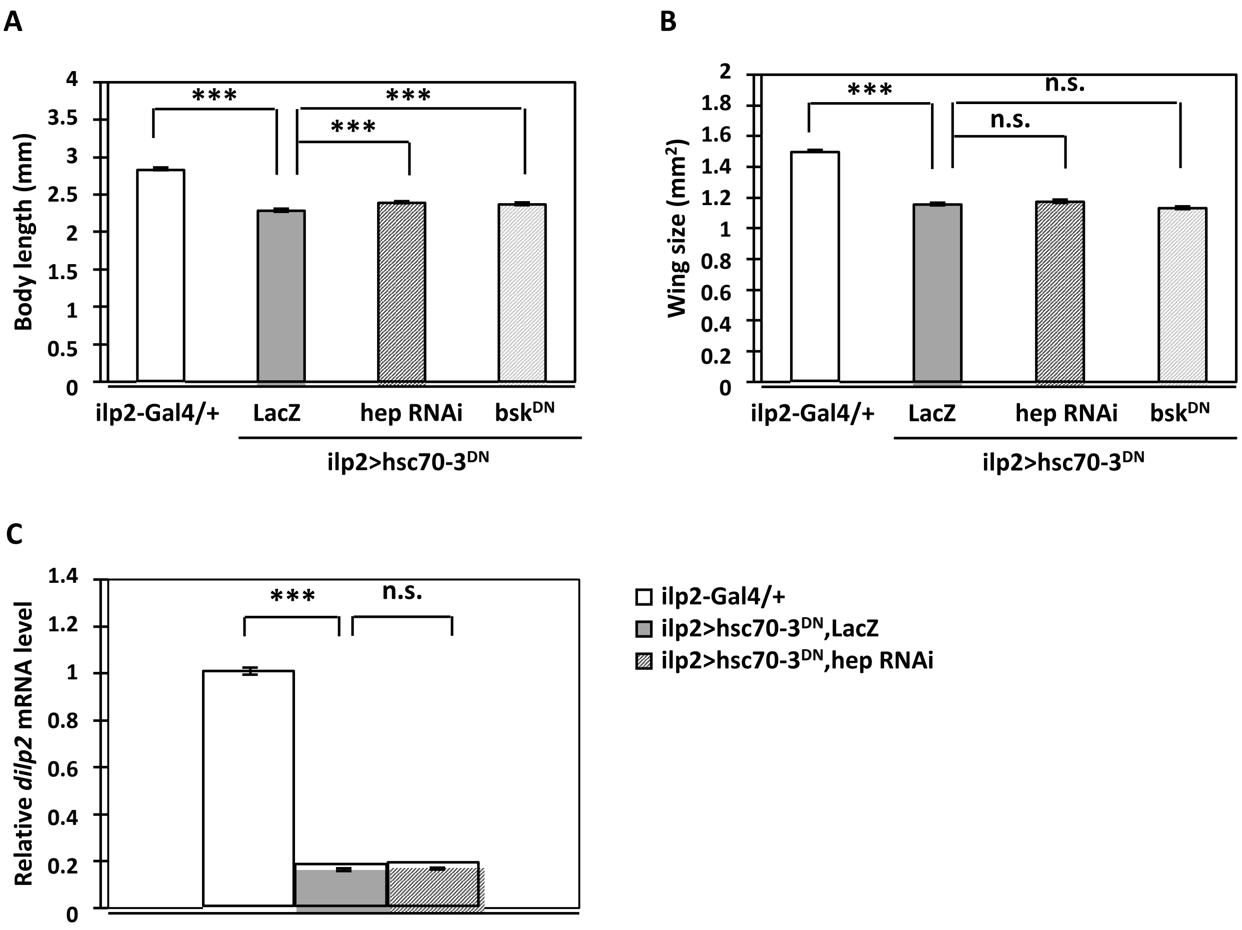

Fig. S4. The effects of inhibiting JNK signalling on ER stress induced-growth phenotypes and reduced levels of *dilp2* mRNA. (A) Quantification of adult body lengths of female flies raised under a condition of ER stress accumulation and inhibited JNK signalling in IPCs. The growth-inhibition phenotype appearing in the body length was slightly rescued by hep depletion or by expressing a dominant-negative form of Bsk

Biology Open • Supplementary information

(n > 30, ***p < 0.001, Student's t-test), whereas the wing area size was not significantly altered (n > 30, n.s.; not significant, p > 0.05, Student's t-test) (B). (C) Relative mRNA-expression levels of dilp2. Total RNA was prepared from adult heads of control (*ilp2-Gal4/+*) females, those expressing Hsc70-3^{DN} in IPCs (*ilp2>hsc70-3*^{DN}, *LacZ*), and these expressing Hsc70-3DN and dsRNA against hep in IPCs (*ilp2>hsc70-3*^{DN}, *hepRNAi*). Relative dilp2 mRNA-expression levels in these adult females were calculated and normalized to the control value (*ilp2-Gal4/+*), which was set to 1.0. Note that no differences in the levels of *dilp2* mRNA were observed between *ilp2>hsc70-3*^{DN}, *LacZ* and *ilp2>hsc70-3*^{DN}, *hepRNAi* flies (n.s.; not significant, p > 0.05, ***p < 0.001, Student's t-test).
